# *Redox Biology* celebrates its first anniversary with over 100 articles, Listing In PubMed and 120,000 downloads with over 230 citations!

**DOI:** 10.1016/j.redox.2014.02.004

**Published:** 2014-03-03

**Authors:** Victor Darley-Usmar, Tilman Grune, Santiago Lamas, Tak Yee Aw

**Affiliations:** a Department of Pathology, University of Alabama, Birmingham, AL, USA; b Institute of Nutrition, Jena, Germany; c Centro de Biología Molecular “Severo Ochoa” (CSIC-UAM), Madrid, Spain; d LSU Health Sciences Center, Molecular and Cellular Physiology, Shreveport, LA, USA

In January 2013, we launched *Redox Biology*, an open access journal as a “new venue for studies in translational, basic and applied research in the fields of antioxidants, cell signaling and redox therapeutics” [1]. A year later, we have achieved our first benchmark as *Redox Biology* celebrates its first anniversary with over 150 published articles and approximately 120,000 downloads. Within the past 12-months, we believe that we have made significant strides as *Redox Biology* steadily gained momentum in recognition and visibility. The average time from submission to first decision is under 2 weeks and papers appear rapidly on line after acceptance and in final form shortly after that (6–7 weeks). Today, this journal is accepted into the bibliographic databases of PubMed Central, Scopus, and Google Scholar which serviced a wide scientific community. We are proud to announce the inclusion of *Redox Biology* in PubMed, and expect shortly afterwards inclusion in JCR—the first step toward acquiring an official impact factor for our journal. Redox biology is having an “impact” with over 230 citations for the 77 articles in volume 1 in google scholar!

One of the many successes of *Redox Biology* has been the graphical reviews, a unique forum for scholarly overviews of the science in our field showcasing the latest concepts, ideas and hypotheses. We were gratified by the overwhelming enthusiastic reception of graphical reviews as evidenced by the impressive number of downloads. The graphical review by Kansanen et al. on the Keap1-Nrf2 pathway [2] is highly popular among our readership, and to date, has over 8000 downloads. Traditional review articles are also well received with Dr. Kalyanaraman’s article on the teachings of basics of redox biology [3] leading the pack with over 3500 downloads. The Editors wish to congratulate these and the many other authors on their outstanding contributions.

At the 1-year benchmark, *Redox Biology* has published over 100 articles on wide-ranging topics in the field of redox biology. As it is our tradition in the past two Editorials, we once again will highlight articles of interest not previously featured. Not surprisingly, three categories emerged as recurrent themes and hot topics, namely, oxidative stress, antioxidant systems and mitochondrial biology.

Oxidative stress remains a highly consistent theme for *Redox Biology*. An article of interest with potential implication for translational research is the review by Ho et al. [4] that addresses recent developments in oxidative stress biomarkers of promise as predictors of prognosis of cardiovascular pathophysiology. Interesting new insights into a beneficial nitrate/nitrite reductase function for xanthine oxidoreductase, an enzyme typically associated with reactive oxygen species generation, were introduced by Cantu-Medellin and Kelley [5]. An original paper by Tseng et al. [6] introduced the provocative notion that low dose radiation-induced oxidative stress can elicit protective radioadaptive effects. On the topic of the lipid peroxidation product, HNE, an original communication of Hardas et al. [7] demonstrated that HNE-modification of lipoic acid and decreased mitochondrial energetics underscores the two-hit hypothesis of Alzheimer’s Disease, while a review by Chapple et al. [8] provided a broad perspective on HNE’s role in redox signaling in vascular pathologies. An important method paper of Weber et al. [9] revealed that the primary antibodies in the ELISA assays are crucial determinants in the quantification of absolute HNE-protein adduct levels. Finally, two back-to-back elegant graphical reviews highlighted the central role that the proteasomal system plays in the maintenance of protein homeostasis through degradation of oxidatively damaged proteins [10,11].

Numerous interesting articles were published on antioxidant systems that covered diverse topics such as Nrf2, dietary antioxidants, glutathione (GSH), and the exciting concept that autophagy, a distinct form of cell death can serve as an important cellular antioxidant mechanism [12]. The transcription factor, Nrf2 was consistently a recurring theme for the journal. Several original research papers dealt with Nrf2 activation and upregulation of antioxidant systems, such as nitric oxide or cytochrome P450 that afforded protection against hypoxia [13] or high-fat diet [14]. Other studies demonstrated notable relationships of Nrf2 activators as potent boosters of GSH and cysteinylglycine [15], or the as yet unresolved competition between Nrf2 and Nrf1 in antioxidant enzyme induction [16]. The investigation into plant polyphenolic compounds as dietary antioxidants has gained prominence in recent years. Recent findings of the antioxidant role of curcumin were discussed in a mini review by Trujillo et al. [17], while an original research paper by Ulasova et al. [18] demonstrated the protective effect of quercetin against dyslipidemia associated cardiac hypertrophy. Apart from its major function as a cellular thiol antioxidant, GSH has emerged as a central player in redox signaling. A possible contribution of GSH to cell cycle regulation was suggested by the finding of a relationship between low GSH and S-phase arrest in the cell cycle [19]. The physiological functions of other small molecules of increasing relevance as pivotal regulators of redox signaling such as NO and H_2_S, were discussed in a graphical review by Kolluru et al. and a more extensive review by Dr. Bailey [20,21]. The intriguing notion that autophagy serves as an important antioxidant function was highlighted in two studies demonstrating that autophagy induction limited hepatic acetaminophen toxicity [22], while autophagy inhibition promoted P450-induced hepatic injury [23]. It is fitting that the 100th article published in *Redox Biology* is an elegant review article by Giordano et al. [12] that addressed the exciting concept that the cellular autophagy-lysosomal system functions is an essential antioxidant pathway.

Studies on mitochondrial biology and function, particularly as related to ROS production continue to be prominent features in *Redox Biology*. Highlighted in this editorial are three original research articles on how changes in mitochondrial morphology or increased ROS generation contributed to vascular pathologies. An interesting role of PDGF-induced mitochondrial fission in the control of vascular smooth muscle cell bioenergetics was described by Salabei and Hill [24]. The next two studies focused on the centrality of  mitochondrial derived ROS in premature vascular smooth muscle cell senescence [25] and pulmonary endothelial dysfunction due to an imbalance in mitochondrial-nuclear cross talk [26]. With implications for site-specific redox (or ROS) signaling within cells, Quinlan et al. [27] provided compelling evidence that the contribution of specific sites to ROS production in the isolated mitochondria depended on oxidation of specific mitochondrial substrates. Without question the role that mitochondria play in health, aging and disease continues to attract growing interest among redox researchers and the search for translational bioenergetic biomarkers is a new and emerging field. Interestingly, cells isolated from patients blood can serve this role and the differential metabolism of circulating platelets and leukocytes suggests they could differentially discriminate between metabolic disorders [28]. In an upcoming issue of *Redox Biology*, a collection of articles related to the diverse functions of the mitochondria in cellular homeostasis and cell survival, will be featured prominently in a special scientific series of articles group into themes.

As we close the page on 2013, we look to 2014 as an expansion year for *Redox Biology*. We fully recognize and appreciate the effort and contributions of many authors, reviewers, editorial board members, and readers to the success of *Redox Biology* this past year. A special thanks also to the journal staff for the rapid handling of the complex task of getting an article into publication and a new author friendly approach to proof correction. We ask repeat authors for your continued support, and encourage contributions from new authors. We, as editors of *Redox Biology*, believe that we have started a quality journal and promise to continue to strive for publications of research excellence in the coming years. We look forward to working with both authors and readers of *Redox Biology* to realize this goal in service to the growing scientific community in the field of redox biology.

Again, many thanks for your support and we wish you the very best for 2014.
